# Essay on Croup, Read before the Atlanta Medical Society, and Published by That Body

**Published:** 1856-03

**Authors:** Eben Hillyer


ATLANTA

Medical and Surgical Journal.

VOL. I.] MARCH, 1856. [No. 7.

### ORIGINAL COMMUNICATIONS.

### ARTICLE I.

*Essay on Croup, read before the Atlanta Medical Society, and published by that body.* By EBEN HILLYER, M. D.

GENTLEMEN:

The subject which I have selected as the theme of my essay, is, as you all know, one replete with interest to the medical man. Every physician has seen it, and witnessed its direful effect upon the infant. There is no disease which excites more terror in the mother than it. She may be wrapt in slumber, worn out and exhausted by the labor and fatigue of her domestic life; but let her sleeping ear catch that peculiar significant sound which she knows to be so characteristic of this fearful disease, and she is awake; she sleeps no longer; she forgets her fatigue; her whole soul is wrapt in solicitude for her offspring.

Croup, Cynanche trachealis of Cullen, Pseudo Membranous Laryngitis, Catarrhal, Croup and Tracheitis, are the most common synonymes. Almost every author adopts a name to suit his own fancy—each one making an effort to supply a name which gives a signification of the pathology of the disease; and as it appears to be more a matter of taste than of sound reasoning, I shall adopt for myself the general term Croup, and consider it divided into two varieties. The Pseudo Membranous and Spasmodic Croup. Laryngismus Stridulus, or spasm of the glottis, is classed among the affections called Croup, by some writers. But I do not think it should be treated of in this connection, as it has peculiar symptoms entirely different from the true Croup. Its mode of attack is different; and it is never attended with that degree of inflammation which exists in Croup. Croup, then, may be defined to be an inflammation of the mucus membrane of the Pharynx and Laryngo tracheal air passages. Pseudo Membranous Croup, the Pseudo Membranous Laryngitis, of Rilliet and Barthez, is in this country a much more rare disease than in Europe; nevertheless, it cannot be called a rare disease even among us. Dr. Condie, in his work upon the diseases of children, says, that during a period of ten years, ending in 1845, there were one thousand one hundred and forty-nine deaths in the city of Philadelphia, from the different varieties of Croup. We may conclude that a large majority of these was Membranous Croup, for the other varieties scarcely ever prove fatal. I have seen, during the past few years, a number of cases of Croup, and I remember to have seen but two cases of this kind. Among the predisposing causes, age is one of the most prominent. Children are more liable to the disease from two or three months after birth, to the tenth or eleventh year. Males are more liable than females. A hale, healthy plethoric infant is sometimes peculiarly liable to an attack. I have found the disease more frequent in the early months of spring. The most frequent among the exciting causes, in my opinion, is the exposure of the infant or child to a cold damp atmosphere. Those who have been much with children, that have constitutional predisposition to disease, as it seems to run in some families, will have noticed that the child is apt, during a cold, damp spell of weather, to get up in the morning with the croupy cough or whoop. Children, convalescent from an attack of serious illness, may be more liable, especially any of the exanthemata. I remember to have seen a fatal case in a child who was convalescent from an attack of scarlatina. Dr. J. F. Meigs says, that it was more frequent in Philadelphia during the years of 1844 and 1845, at which time measles and scarlatina were prevailing to an unusual degree.

*Anatomical Lesions.*—The mucous membrane of the larynx and trachea, and often the pharynx, is found covered with a false membrane—a tenacious, yellowish-white material. It is fitted in these air passages accurately in bad cases. Sometimes it extends even down into the Bronchial tubes.

I assisted Prof. Mutter in performing the operation of tracheotomy, in an hopeless case two years ago. When the opening was made into the trachea, a considerable quantity of this substance was thrown out in the efforts for free respiration. The case proving fatal, Dr. M. requested a post mortem; which I assisted in making. We found the false membrane extending throughout the larynx, the trachea, and even down into the bronchial tubes; some of which we were able to detach, and pull out in perfect cylindrical pieces. The inflammation had, in this case, extended down into the parenchyma of the lungs, as is often the case in those which terminate fatally. The mucous membrane of the larynx and trachea, we found, after removing the extended membrane, to be of a violet color.

The false membrane sometimes extends into the pharynx, covering the tonsil glands. In fact, the inflammation frequently has its commencement in the pharynx.

Dr. Cheyne, the author of the article upon Croup, in the Cyclopædia of Medicine, compares the tubular appearance of the removed false membrane, to pieces of macaroni boiled in milk.

*Symptoms and Course.*—The child attacked with Croup, often has catarrhal symptoms for several days previous to the accession of the severe attack. It exhibits evidences of indisposition; it is irritable, cheeks flushed, restless, and in the morning, is apt to have the cough which is so characteristic of Croup. It is a peculiar ringing cough. It cannot be described, yet one who has once heard it, will never mistake it. As I said before, there is no sound made by the infant, which will enlist the attention of the mother sooner than it. Dr. Cheyne gives it a name—the Tussis Clangosa—saying that it sounds like the child had coughed through a brazen trumpet. The attack is not always ushered in with these prodromes, for we often find the child awakened out of its sleep, with a violent attack, without any warning whatever. As the disease progresses, the voice is changed, and has a peculiar twang about it, something like the cough. The hoarseness and alteration of the voice is dependent upon the severity of the attack upon the larynx and the spasm of its muscles. The respiration now becomes very laborious, and much more frequent. Dr. Meigs has seen it forty-eight respirations to the minute. The whole force of the respiratory apparatus is required to keep up this function. The little sufferer appears certainly to be on the verge of suffocation. The air whistles and hisses through its passages with a sibilant rale. This is caused by the exuded membrane obstructing the ingress and egress of the air. In the cases which I have seen, the face of the child gave evidence of extreme anxiety, of terror; and its face was of a purplish blotched hue. There is no expectoration early in the disease; when it is exhibited, we find portions of false membrane mixed with mucus, and we are recommended to put the expectorated matter in water, and the membrane is more easily detected. Sometimes it is coughed up in the tubular form as before described. When the disease has reached its acme, there are considerable febrile symptoms. The pulse goes up 130, and some have observed as high as 150 beats to the minute. If there is much membranous expectoration, we may hope for the disease to terminate favorably. The hoarseness will gradually become less, and all the symptoms abate, and the child be well in from two to ten days, according to the severity of the attack. If the case is to be fatal, the symptoms become more aggravated; the respiration more labored; the intercostal muscles and diaphragm doing their utmost to force the air through the occluded and constricted air passage; the inflammation often extending down into the lungs, and making a pneumonia. There is the most distressing orthopnœa, and finally, the little sufferer is relieved of its great distress by death.

In regard to the prognosis, all writers agree that Pseudo Membranous Croup is a most dangerous and fatal disease. I have been taught it is the most fatal disease to which a child is subject. I find from my books, that Guersent saw an hundred cases of Spasmodic Croup, and none were fatal; of ten cases of Pseudo Membranous, two were fatal. M. Terrand observed sixty cases at Aix-La-Chapelle, not one of which escaped. It is very important that we should make an early diagnosis of this variety from the more common and far less fatal form of Croup, viz.: Spasmodic Croup, Catarrhal Croup, of

Wood, Spasmodic Laryngitis, of others. This form is by far the most common; it occurs so frequently in some families, that the parents of the children are always prepared for it, having seen so much of it that they understand the routine treatment perfectly, and are never without the Compound Syrrup of Squills, upon which they chiefly rely. It often commences with coryza a day or two previous to the final attack; and a wheezing cough, something like the cough ushering in measles. Sometimes the little patient is fretful, and slightly feverish, showing from its general appearance, that it is suffering from some derangement. After these symptoms have prevailed for a short time, or as is very frequently the case without any premonition whatever, the child is attacked with much the same kind of a paroxysm, as in the more grave form, although arising from a different pathological cause. That the diagnosis of this disease may be more clear, I will give the following table of difference of Rilliet and Barthez, as modified by Dr. J. F. Meigs:

*Spasmodic Croup.*—Begins with coryza and hoarse cough, or more frequently by a sudden attack of suffocation in the night, fauces natural, or merely a slight redness, as in simple angina.

*Pseudo Membranous Croup.*—In epidemic form, begins as Pseudo Membranous Angina; in Sporadic form, invasion of slight hoarseness for a day or two. There is fever, increase of hoarseness, with hoarse croupal cough. In half the cases, Pharyngeal exudation, and a little later, paroxysm of suffocation.

*Spasmodic Croup.*—After the paroxysm, the child seems well; the fever disappears, or is very slight; voice natural or slightly hoarse, not whispering.

*Pseudo Membranous Croup.*—The fever continues; stridulous respiration; prolonged and difficult expiration; cough hoarse and smothered; voice hoarse and whispering.

*Spasmodic Croup.*—If the paroxysm returns, it is during the following night, and it is less severe. The hoarseness disappears; the cough becomes loose and catarrhal.

*Pseudo Membranous Croup.*—Dyspnœa and suffocation increased; the voice and cough is smothered or extinguished; stridulous respiration persists.

*Spasmodic Croup.*—Duration seldom more than three days.

*Pseudo Membranous Croup.*—Duration seldom less than five days; the hoarseness continuing for several weeks.

*Spasmodic Croup.*—Very rarely fatal.

*Pseudo Membranous Croup.*—Fatal in a majority of cases. In considering the treatment, most writers have confounded the two principal varieties together. I shall first notice that of the grave form. I am not decidedly of the opinion, from what has been my observation, that any treatment we may adopt, will be of little avail. The course decided upon, should be active and continued, until the inflammation is checked.

Blood-letting is one of the most powerful means we have of combating any of the phlegmasia, and I find there is some diversity of opinion in regard to its use. Dr. Cheyne is in favor of depletion, both general and local. Wood says, that it is more effectual in Spasmodic Croup. From Dr. J. F. Meigs' work, I find that Guersent asserts that depletion does not arrest this inflammation; that the disease continues, and terminates fatally, notwithstanding the most active use of the lancet. Valleix, with Brettonneau, is of opinion, that bleeding has no curative means, and does not obviously arrest the progress of the disease. On the other hand, Dewees says, "that it is the remedy upon which our principal reliance should be placed." Condie says, "it is the most effectual means of arresting the disease; and the practitioner who places his hopes upon any other means, and neglects this, cannot flatter himself upon his success in its management." I take a midway view as to this measure. If the pulse is full and bounding, the fever rising, the child plethoric and of full habit, I should certainly bleed it. In diminishing the volume of blood, aside from its specific effect in counteracting the inflammation, it gives great relief to the respiration, by the lessened quantity of blood sent to the lungs. Then, when we bleed, there is not the danger of pneumonia. If the patient is weak and thin, or should the disease have progressed to a considerable extent, I should not bleed. The use of emetics is admitted by all writers. They all agree that more reliance is to be placed in them than in any other means. In Membranous Croup, they are beneficial by their sedative effect in bringing down the inflammation. Then the ejection of the false membrane is greatly facilitated by their use. M. Valleix relied upon Tartar Emetic and Ipecac in thirty cases, of which fifteen were cured. To a child two years old, I should give from ¼ to ½ gr. of Tartar Emetic, and from three to five of Ipecac, every twenty minutes, until full vomiting is excited. My former instructor, Dr. C. D. Meigs, recommends powdered alum, as the most reliable emetic. I have heard him eulogize this remedy very highly; having used it for many years, he confidently speaks of its efficacy as a prompt agent. He gives it in the spoon full doses—the powder mixed with honey or syrup, and repeats every twenty minutes, until vomiting is excited. The second dose rarely has to be given. He has succeeded with the alum, when Tartar Emetic had failed to excite emesis.

Dr. Logan of this place informs me that he has frequently used the alum, and that in his hands, it gave entire satisfaction. I have never used it myself, but from the high authority in its favor, I feel safe in recommending it to a favorable notice. After emetic action, I should give calomel in one or two grain doses, with ¼ gr. Tartar Emetic, every two hours. I have great confidence in the calomel, in the grave form of Croup. In its specific action upon the blood, in diminishing the fibrin, it must be of service, for from this material, the fibrinous exudation is formed. Some practitioners have pushed this agent to an extent wholly unwarrantable. After its effects are fully manifest upon the system, it should be discontinued. This remedy should be persevered in until there is an abatement of the paroxysm, or until we see that all effort is futile.

In Spasmodic Croup, little else is necessary than the administration of the emetic. The warm bath will greatly facilitate its action, in producing a sufficient degree of relaxation to overcome the spasm. It is very well to subject the infant to warm bath in either case, as there is spasm of the muscles of the larynx in both cases. The Seneka snake root is sometimes given. It forms one of the ingredients of the compound syrup of squills, of Dr. Cox. This is used more is Spasmodic Croup than any other preparation, and I have no doubt with more success. It has the emetic property of the tartar emetic combined with the expectorant of the seneka and syrup of squills. It is usually given in doses of from 20 to 30 gtt., until it vomits. There are a number of remedies recommended by authors in high terms; and I think it useless to refer to them, as all the indications required can be carried out by the agents referred to. As a dernier resort, Tracheotomy has been often tried. The French, from their statistics, have been more successful with it than any other nation. With us, the operation is in no high favor.

